# Prevention and control of schistosomiasis in the Philippines from a health education perspective

**DOI:** 10.3389/fpubh.2025.1558564

**Published:** 2025-04-09

**Authors:** Xiaodong Yao, Yihan Chen, Keda Chen, Lijun Lin, Jiangyue Zhong, Chaojun Shan, Mingcheng Liu, Xintong Chen, Yijie Zhang, Hongyu Li

**Affiliations:** ^1^School of Marxism, Hangzhou Medical College, Hangzhou, China; ^2^Key Laboratory of Artificial Organs and Computational Medicine in Zhejiang Province, Shulan International Medical College, Zhejiang Shuren University, Hangzhou, China; ^3^School of Basic Medicine and Forensic Medicine, Hangzhou Medical College, Hangzhou, China

**Keywords:** schistosomiasis, *Schistosoma japonicum*, health education, Philippines, prevention strategies

## Abstract

Schistosomiasis, second only to malaria, poses a significant threat to many regions worldwide, particularly tropical and subtropical areas. The Philippines, located in a tropical region, has long suffered from the serious public health hazards of schistosomiasis. We recognize that, besides direct snail control and mass drug administration, education plays a crucial role, either directly or indirectly, in the prevention and control of schistosomiasis. Therefore, this paper delves into the current status of schistosomiasis in the Philippines, the evolving strategies for prevention and control, and the significance of these efforts, with a particular focus on analyzing the impact, achievements, and challenges of educational interventions in schistosomiasis control. This detailed analysis aims to provide a comprehensive perspective on the overall progress and challenges of schistosomiasis prevention and control in the Philippines.

## 1 Introduction

Schistosomiasis is a parasitic disease caused by schistosomes, also known as a neglected tropical disease, and is one of the most devastating parasitic diseases, second only to malaria (1). The transmission of schistosomiasis is highly correlated with sanitary conditions, so the economic level directly affects the infection and mortality rates of schistosomiasis, its distribution is closely aligned with economically underdeveloped areas, with major endemic regions including Africa, the Middle East, South America, the Caribbean, and Southeast Asia (2). Currently, more than 220 million people and a large number of animals are infected with schistosomiasis (3). Globally, there are various pathogenic schistosomes, with their scope of influence distributed differently yet overlapping to some extent (4). Among these, the most impactful and severe species are *Schistosoma mansoni*, *Schistosoma haematobium*, and *Schistosoma japonicum*. Other species, such as *Schistosoma intercalatum* and *Schistosoma mekongi*, were previously considered less impactful. However, their significance is increasing due to climate change, which is believed to be driving their emergence in new regions (5).

The Philippines is one of the major endemic areas for *Schistosoma japonicum* (6, 7). Humans are the definitive hosts for *Schistosoma japonicum*. In addition to humans, many mammals can serve as reservoir hosts for *Schistosoma japonicum*, including cattle, pigs, dogs, cats, and rodents (8, 9). Water buffalo involved in agricultural production are the most important reservoir hosts, with a prevalence rate of approximately 80% for Schistosomiasis japonica in Filipino water buffaloes (10). Schistosomiasis japonica belongs to hepatointestinal schistosomiasis and ectopic schistosomiasis. In addition to causing severe public health problems, schistosomiasis has also significantly impeded socioeconomic development (11).

In the 1930s, the importance of health education in controlling schistosomiasis was acknowledged, although control efforts primarily focused on eliminating intermediate hosts at that time. With the widespread use of oral antiparasitic drugs in the late 1970s and the emergence of more rapid and efficient field parasite detection techniques, the focus shifted from solely snail eradication to comprehensive control measures. The extent of schistosomiasis spread is closely related to four factors: the level and extent of water contamination with schistosome eggs, the quantity and characteristics of intermediate host, the degree of human exposure to contaminated water and protective measures, and the lifespan of schistosome parasites in the human body (12). When the quantity of intermediate host decreases to a certain level, the remaining hosts become more scattered and potentially, significantly increasing detection challenges and complicating eradication efforts (13). Therefore, altering human behavior is crucial in preventing and controlling schistosomiasis (12). If humans can actively reduce exposure to contaminated water sources and use chemical drugs as directed by medical advice, then schistosomiasis transmission can be more effectively controlled (4).

The Philippines has made notable economic progress in recent decades, yet the income gap remains significant. The poor, often lacking good sanitary conditions and having limited knowledge of health and hygiene, contribute to the persistence of schistosomiasis. With the increasing global awareness of health, the role of health education in preventing and controlling parasitic diseases is gaining more attention (14). This paper begins with an analysis of the current status of schistosomiasis in the Philippines, introduces the life cycle of *Schistosoma*, and delves into the development of prevention and control strategies in the country, with a particular focus on the impact, achievements, and challenges of educational interventions in schistosomiasis control. It aims to systematically reveal the overall progress and key challenges in the prevention and control of schistosomiasis in the Philippines, providing a scientific foundation for future research and practice.

## 2 Methods

This study is a narrative literature review, aiming to comprehensively describe and summarize existing research on the prevention and control of schistosomiasis in the Philippines. Adhering to the PRISMA (Preferred Reporting Items for Systematic Reviews and Meta-Analyses) guidelines for literature screening and inclusion. The literature search was conducted up to March 2025, with data sources including PubMed and Google Scholar. The search strategy incorporated both keywords and subject terms, focusing on the following: (Schistosomiasis), (*Schistosoma japonicum*), (Philippines), (schistosomiasis japonica control), (Health education, water, sanitation and hygiene services), (PCR and loop-mediated isothermal amplification (LAMP)), (Elimination Program), (Mass Drug Administration (MDA)), (Praziquantel), (Neglected Tropical Diseases).

The search terms were combined as follows: ((schistosomiasis japonica control) AND Philippines), (schistosomiasis health education), ((Neglected Tropical Diseases) AND (Schistosomiasis)), ((Praziquantel [Title]) AND (schistosomiasis)), (Mass Drug Administration (MDA) for Schistosomiasis), ((Schistosomiasis Control) AND (Elimination Program)), ((*Schistosoma*) AND (PCR and loop-mediated isothermal amplification (LAMP))), ((water, sanitation and hygiene services) AND (schistosomiasis)). Additionally, citation searching and manual screening of references from relevant review articles were performed to identify further studies that met the inclusion criteria.

The inclusion criteria for this study were as follows: (1) Studies exploring “From a health education perspective, the prevention and control of schistosomiasis in the Philippines”; (2) Studies related to schistosomiasis prevention and control strategies, intervention measures, or influencing factors; (3) Multiple types of literature, including quantitative studies, qualitative studies, reviews, and policy analyses; (4) Articles published in English.

The exclusion criteria included: (1) Articles unrelated to the research topic; (2) Duplicate literature; (3) Non-English publications without available translations; (4) Literature for which the full text is unavailable.

**Figure 1:**
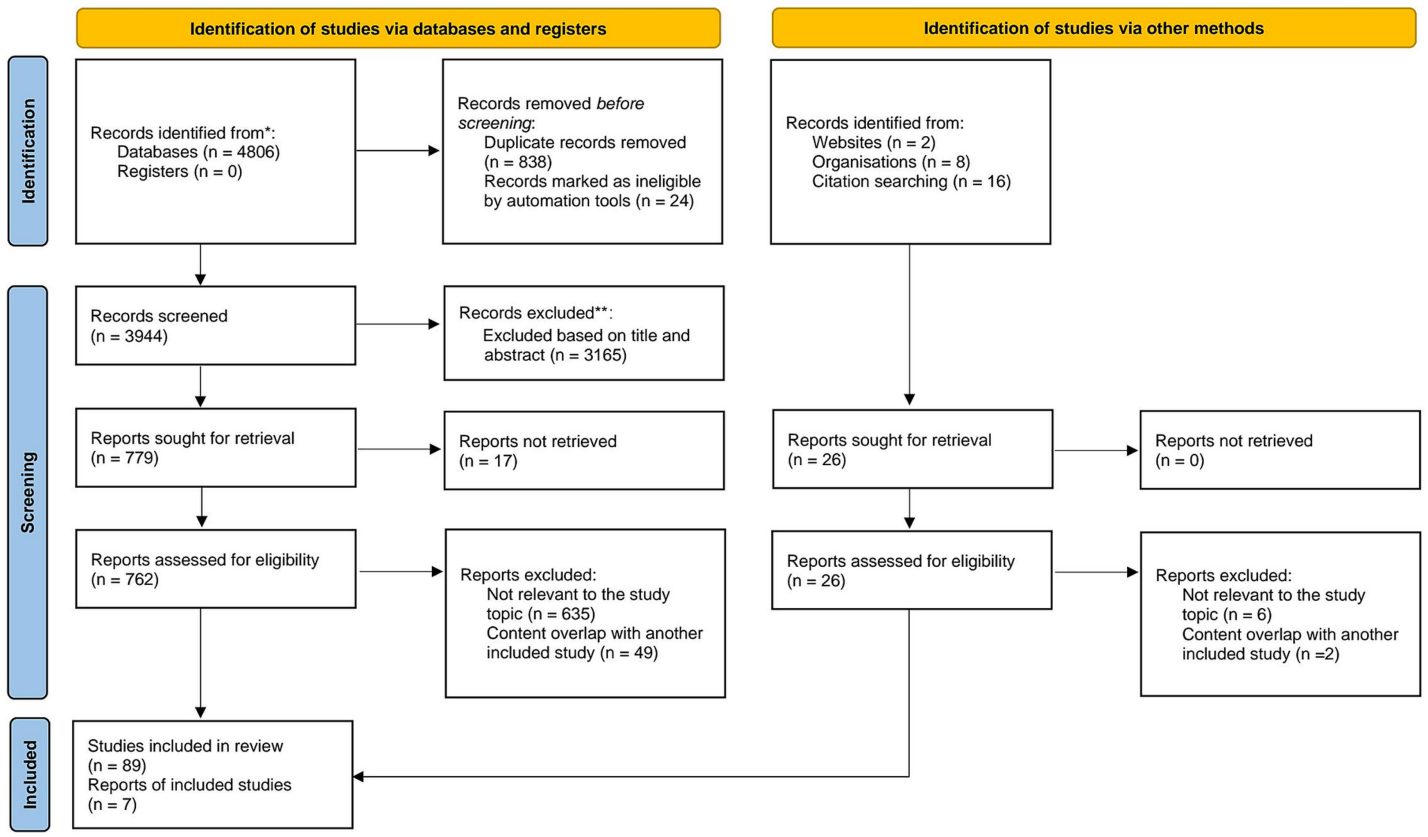
PRISMA flowchart for literature search. Source: Page MJ, et al. BMJ 2021;372:n71. doi: 10.1136/bmj.n71. This work is licensed under CC BY 4.0. To view a copy of this license, visit https://creativecommons.org/licenses/by/4.0/.

This study followed the PRISMA framework for literature screening (Figure 1). The database search was primarily conducted in PubMed, with Google Scholar as a supplementary source. Initially, 4,806 articles were retrieved. After removing 838 duplicates, an automated screening tool (EndnoteX21) excluded 24 articles with incomplete information. This left 3,944 articles for initial screening. Titles and abstracts were reviewed, resulting in the exclusion of 3,165 irrelevant studies. Ultimately, 779 articles were selected for full-text review. Among these, 17 articles could not be accessed and were excluded. A detailed eligibility assessment was then conducted on 762 articles. After reading the full texts, 635 irrelevant studies were excluded, and an additional 49 studies were removed due to their similarity to already included articles. Studies identified through other methods included: Websites (2 articles, of which 2 were excluded after further evaluation), Organizations (8 articles, of which 1 was excluded after evaluation), Citation searching (16 articles, of which 5 were excluded). Finally, 89 studies meeting the inclusion criteria were included, along with 7 research reports.

## 3 Current status of schistosomiasis in the Philippines

**Figure 2:**
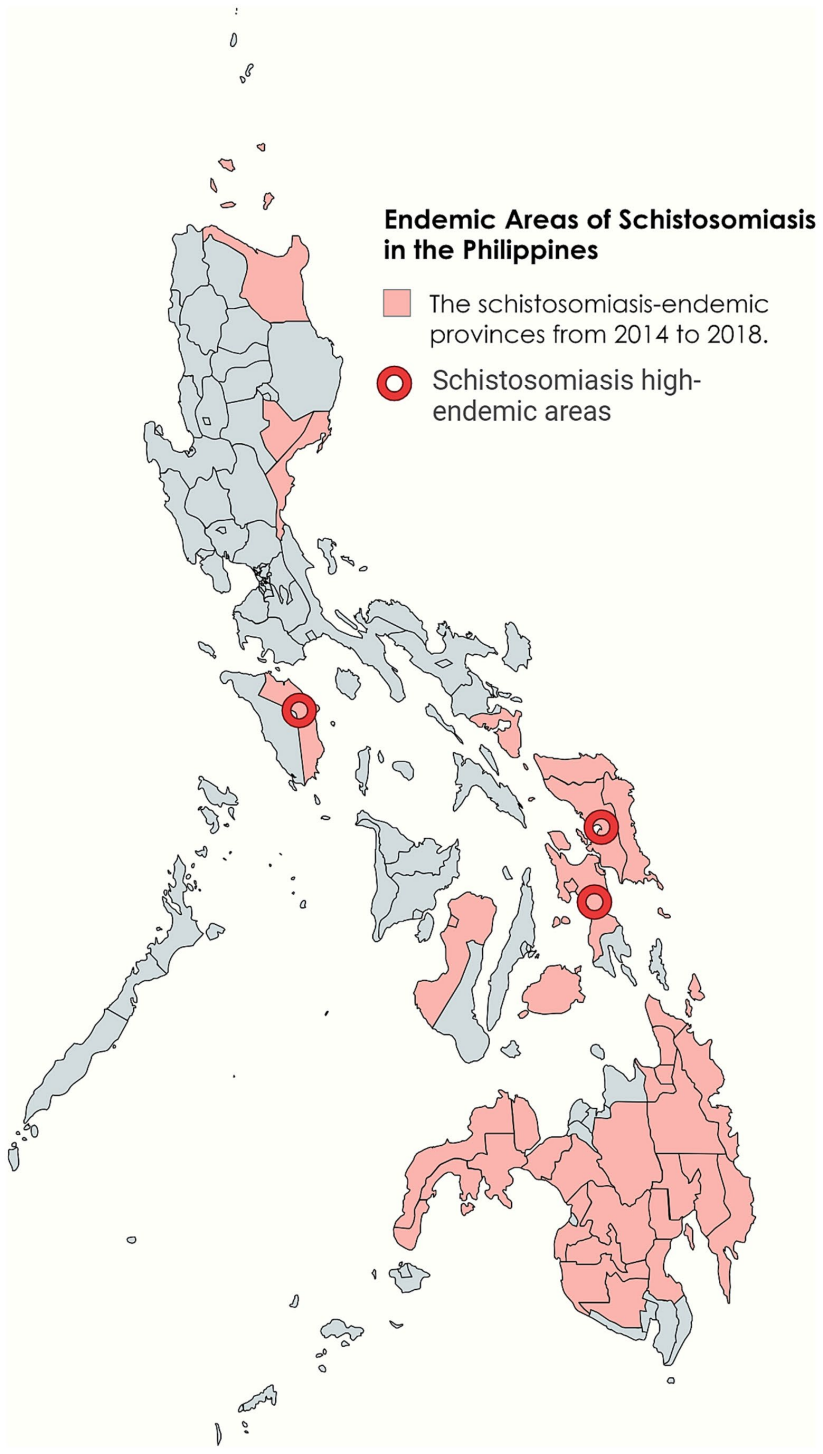
Endemic areas of schistosomiasis in the Philippines. Figure was created with BioRender.com and MapChart.net.

The Philippines is located in Southeast Asia, bordered by the South China Sea to the west and the Pacific Ocean to the east. It is an island nation composed of over 7,000 islands and island groups (15). The country has a monsoon-type tropical rainforest climate, characterized by high temperatures and humidity, and abundant rainfall, with an average annual temperature of 27°C (16). The major regions of the Philippines do not have a distinct dry season, which means the transmission of schistosomiasis occurs year-round (17). Unique geographical condition and climate have created ideal conditions for the transmission of schistosomiasis in the Philippines (18) (Figure 2).

Schistosomiasis is widespread in 28 provinces across 12 geographical regions on the three major island groups of Luzon, Visayas, and Mindanao. The Eastern Visayas, particularly the islands of Leyte and Samar, are the main endemic areas (19, 20). As various factors such as climate and population change, the prevalence of schistosomiasis also changes. In recent years, approximately 12 million people in the Philippines are at risk of schistosomiasis infection, with about 2.5 million directly infected and over 800,000 diagnosed with active schistosome infections (19). However, there have been contradictory reports about the prevalence of schistosomiasis in the Philippines. Regional reports usually show higher infection rates, while nationwide surveys that include non-endemic areas may lead to lower estimates. This variation in data could be due to different data sources and research methodologies (21).

Due to differences in race, age, gender, and occupation, patients may have varying probabilities of exposure to endemic water sources and, consequently, different probabilities of schistosomiasis infection (22). In low prevalence environments, the infection rate and incidence of schistosomiasis can be effectively reduced (23). Age is a contributing factor to changes in infection rates, which are directly determined by exposure levels, water exposure tends to decrease with age (24). Nutritional status in children and young adults has a certain correlation with schistosomiasis, with infected individuals generally exhibiting lower height, weight, and BMI (25). Especially in children, schistosomiasis can lead to adverse effects such as anemia, malnutrition, and cognitive decline (26, 27). Attributed to sociocultural or behavioral factors, men tend to have higher infection rates than women. Immunologically, men and women exhibit different strengths in humoral immune response against schistosome invasion (22). Schistosomiasis japonica can be classified into the four types based on the patient’s disease course and the characteristics of the affected areas (28). Acute schistosomiasis has a short incubation period and commonly presents symptoms such as fever, rash, and diarrhea. Chronic schistosomiasis has a long course, with some cases lasting 10–20 years, characterized by hepatitis or colitis. Advanced cases may exhibit symptoms like splenomegaly and ascites. Ectopic schistosomiasis refers to the infestation of schistosome adult worms or eggs in tissues or organs outside the portal vein system (29).

## 4 General life cycle and transmission

**Figure 3:**
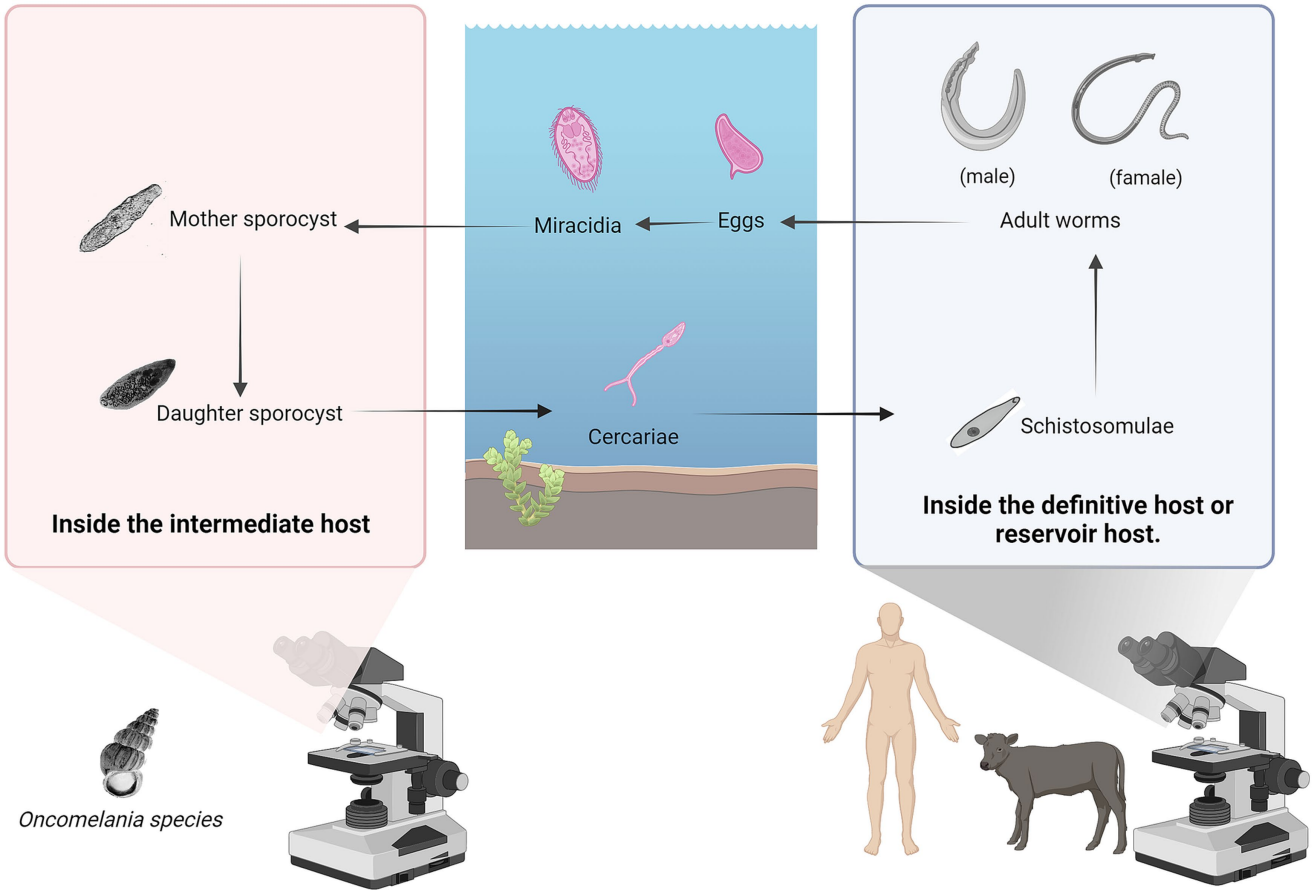
The life cycle of the *Schistosoma japonicum*. Its life cycle includes seven stages: egg, miracidium, mother sporocyst, daughter sporocyst, cercaria, schistosomulum, and adult worm. Figure was created with BioRender.com.

*Schistosoma japonicum* was first discovered and named by Japanese scientists. Its life cycle includes seven stages: egg, miracidium, mother sporocyst, daughter sporocyst, cercaria, schistosomulum, and adult worm. The adult worm lives in the portal vein system of humans or other mammals (the definitive host). After mating, the female schistosome produces a large number of eggs. Due to the pressure of blood flow, the eggs deposit in the liver and intestinal wall tissues of the final host. The eggs then enter the intestinal cavity along with the damaged tissue and are excreted from the body through the feces of the final host (30). In suitable hatching conditions in water, fertilized eggs hatch into miracidia, they swim in water, seeking intermediate hosts. After infecting the intermediate host, the miracidia develop into mother sporocysts within 48 h. The mother sporocysts reproduce asexually to produce and release daughter sporocysts, cercariae mature within the daughter sporocysts and are released, escaping through the opening in the snail’s operculum. These infectious cercariae swim freely in the water, seeking an opportunity to infect the definitive host, thus beginning a new cycle (31) (Figure 3).

## 5 Strategies for schistosomiasis control in the Philippines

**Figure 4:**
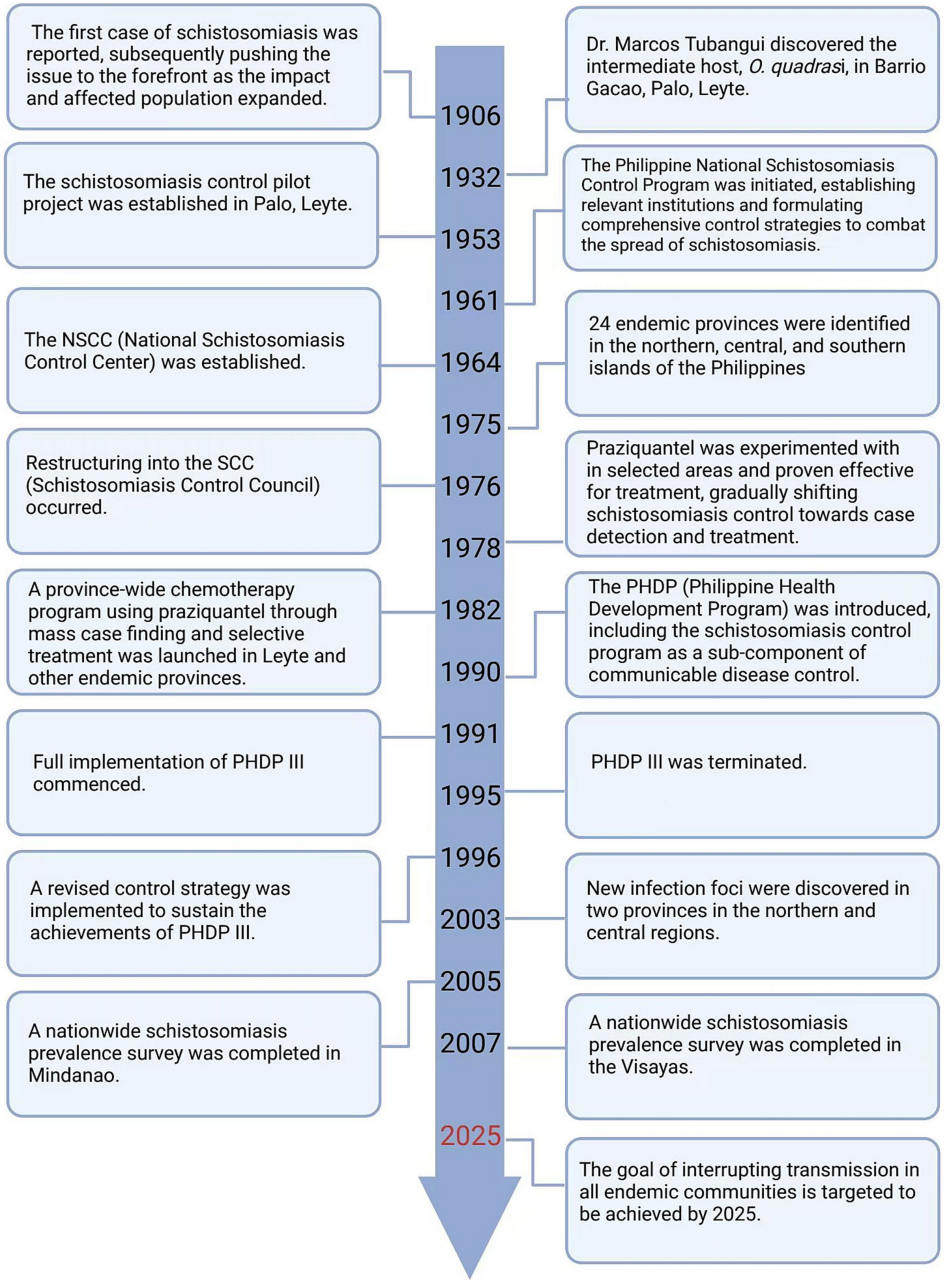
The timeline of changes in the strategies for the prevention of schistosomiasis in the Philippines. Figure was created with BioRender.com.

The records of the first case of schistosomiasis in the Philippines date back to 1906, as the impact expanded, the issue of schistosomiasis has been brought to the forefront (11). In 1961, the Philippine National Schistosomiasis Control Program was launched, establishing relevant institutions and creating comprehensive control plans to combat the spread of schistosomiasis (11). In the early stages of control, there was a lack of overall understanding of schistosomes and a lack of safe and effective drugs. The most direct and effective prevention and control measure was to interrupt the parasite’s life cycle, specifically by eradicating snails. However, implementing this measure is costly and difficult to conduct on a large scale for an extended period of time. In 1978, praziquantel was tested in selected areas for treatment and was found to be effective. The control of schistosomiasis gradually shifted towards the detection and treatment of cases (11). As prevention and control efforts continue and the distribution of the disease changes, the structure and plans of management departments are adjusted based on the actual situation, and inter-departmental cooperation is becoming increasingly close. Given the complex issue of schistosomiasis, experience tells us that a single-level solution is not feasible; a comprehensive approach is required to address this multifaceted problem (14) (Figure 4).

The Schistosomiasis Control and Elimination Program (SCEP), led by the Department of Health (DOH), focuses on implementing the following measures: (1) Preventive Chemotherapy and infection Control; (2) Transmission Control; (3) Public-Private/ P2P Partnerships; (4) Advocacy and social mobilization; (5) Monitoring and evaluation (32).

The overall goal is: (1) To reduce Annual Parasite Index (API) to <1% of the population to established endemic barangays/municipalities. (2) To strengthen program performance by empowering LGUs through a community based implementation (32). The plan aims to eliminate the transmission and incidence of schistosomiasis in all endemic villages by 2025, making the achievement of this goal somewhat challenging (33).

**Figure 5:**
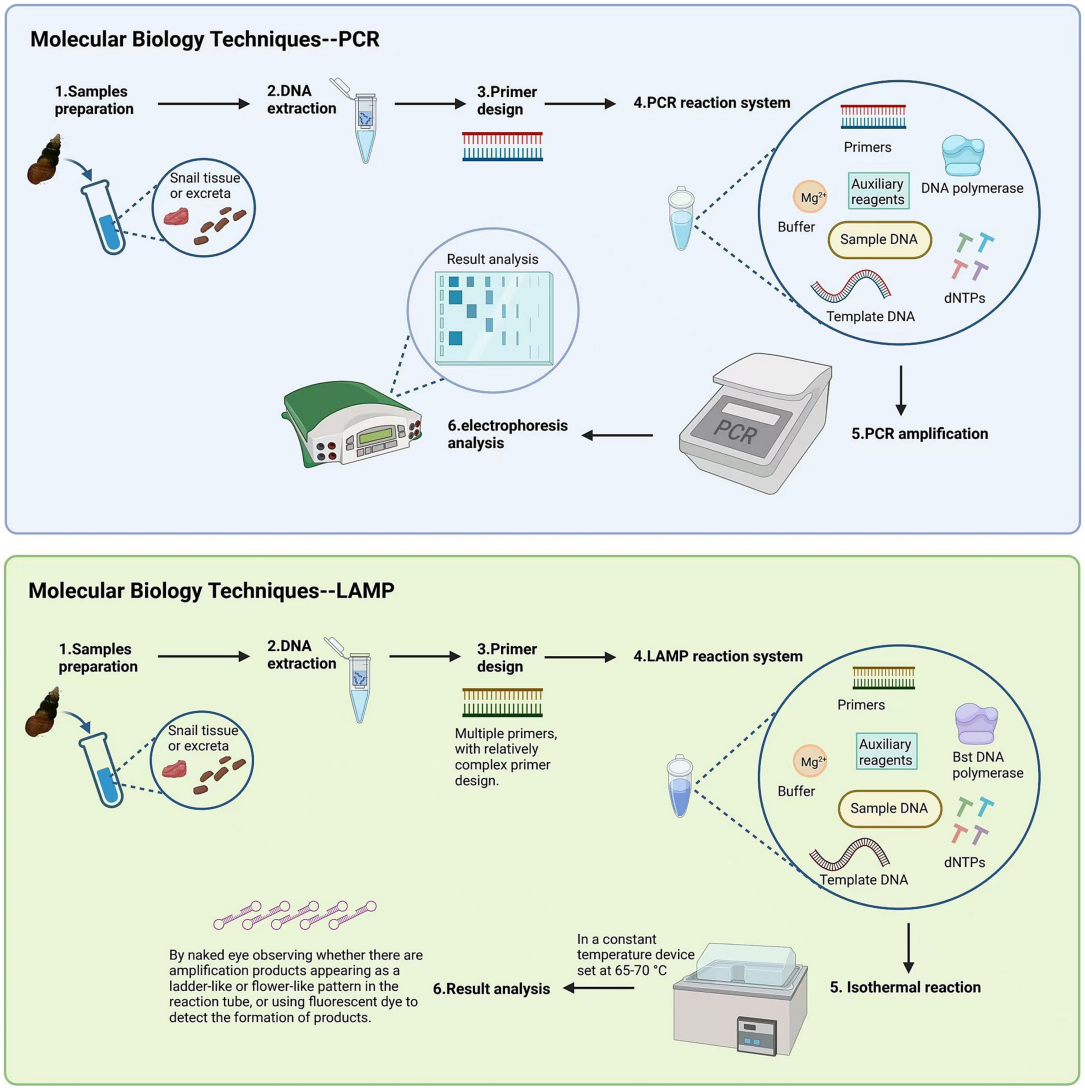
The comparison of detection methods between LAMP and PCR. Figure was created with BioRender.com.

Developing overall strategies, understanding disease trends, and assessing the effectiveness of control measures all rely on epidemiological investigations of schistosomiasis. For developing countries with limited funding, the cost of some diagnostic technologies is high, making it difficult to widely implement them in practice (34). In addition to monitoring the number of infected individuals, testing whether snails are infected with miracidia is extremely important for schistosomiasis control efforts. Traditional detection methods have clear drawbacks and no longer meet current field detection needs. Polymerase chain reaction (PCR) and loop-mediated isothermal amplification (LAMP) are currently the main detection methods. The sensitivity of both methods is similar, but PCR requires complex equipment and a high level of technical skill (35). LAMP has high sensitivity and specificity and does not depend on complex specialized equipment. The process only requires a constant temperature instrument, and the amplification product is a colored precipitate, allowing visual confirmation of whether amplification has occurred without the need for special equipment. This makes it suitable for field applications (36). Detection methods continue to evolve, such as recombinase-aided amplification (RAA), which not only simplifies operations but can also be used for bulk testing of mixed snail samples (37). By combining detection data and using RS and GIS technologies, a model can be established to reflect the spatial distribution of schistosomiasis in the Philippines and its influencing factors. This provides strong support for the subsequent development of precise control strategies (38) (Figure 5).

Currently, praziquantel is the first-line treatment for all forms and age groups of schistosomiasis. Its recommended dosage varies according to age and weight, with WHO recommending a dosage of 40 mg/kg. Previously, the dosage used in the Philippines was 60 mg/kg. However, studies have shown that a higher dosage of 60 mg/kg of praziquantel does not offer significant therapeutic advantages in treating intestinal schistosomiasis caused by *Schistosoma mansoni* or *Schistosoma japonicum* compared to the standard dosage of 40 mg/kg. Therefore, the Philippines has shifted from using a dosage of 60 mg/kg to 40 mg/kg (39). Praziquantel’s side effects are often mild and short-lived. Clinical studies have found that the frequency and severity of side effects are directly related to the intensity of infection (40).

Currently, there is no commercially available vaccine for human schistosomiasis. Candidate human schistosomiasis vaccines under research are in different stages of preclinical and clinical development (41). As *Schistosoma japonicum* accounts for a very low proportion of total schistosomiasis cases, these vaccine candidates primarily focus on *Schistosoma mansoni* and *Schistosoma haematobium*, which have higher case counts. Further research on other schistosomiasis can also provide insights for *Schistosoma japonicum* (42). In regions where schistosomiasis caused by *Schistosoma japonicum* is endemic, animals play a key role as reservoir hosts in the transmission of schistosomiasis due to its zoonotic nature. For instance, cattle, as a reservoir host, can cause up to 90% of environmental contamination with parasite eggs (43). With advances in technology, the widespread adoption of modern mechanized farming techniques can replace the work of many cattle and reduce the rearing of intermediate host cattle, indirectly reducing the spread of schistosomiasis (44). From a vaccine development perspective, researching animal vaccines is essential. Many candidate vaccines have been developed to interrupt *Schistosoma japonicum* transmission (45). In summary, reducing the rearing of cattle in endemic areas and continuing to vaccinate them is a key strategy (20).

Beyond mass drug administration (MDA), controlling the transmission of schistosomiasis involves multiple factors (46). The Philippine government has taken various measures to control the population of intermediate host, including but not limited to the use of chemical molluscicides, biological control, and improvements in environmental sanitation (11). Notably, niclosamide is the commercially available molluscicide currently recommended by WHO, as it is effective at killing snails and has relatively low environmental toxicity (47). Many new molluscicides have shown promising results in experimental conditions, with potential for future large-scale use. For example, four derivatives of salicylanilide esters (48).

One survey found that the level of knowledge about schistosomiasis and attitudes toward disease prevention among study participants were highly correlated with their rate of infection, individual cognition and sociocultural factors play a guiding role in the control of schistosomiasis. Therefore, the future direction of public health efforts should focus on fostering preventive behaviors in residents and instilling community beliefs in disease prevention. This approach aims to increase the impact of disease control programs in endemic communities by considering the sociocultural and behavioral context (4).

## 6 The crucial role of health education

Education plays an indispensable role in the prevention and control of schistosomiasis. A meta-analysis showed that after controlling for confounding variables, and following an experiment duration of 2 years, the group that received health education saw a decrease in the overall prevalence of *Schistosoma japonicum*, while the control group saw no significant change. This suggests that receiving health education may have a significant impact on schistosomiasis (49). Before 1930, health education was largely neglected, and for the next 40 years, it also largely overlooked the social context. It wasn’t until the development and application of praziquantel, along with snail eradication methods to reduce schistosomiasis transmission, that the focus shifted to controlling incidence rates. This was when people began to realize that controlling the incidence of schistosomiasis is inseparable from education (12). Health education was subsequently incorporated into Primary Health Care (PHC) (50).

Patients struggle to afford treatment, education levels are low, and awareness is limited, leading people to view it as a “commonplace” disease and largely ignore the dangers of schistosomiasis (51). Although lower education levels and passive attitudes are key factors in disease transmission (52). The relationship between education levels and reducing prevalence rates is not absolute, as prevalence also depends on other factors (52). These factors are often at the societal level, such as economic development, healthcare investments, the emphasis on controlling specific diseases by community and nation al health planners, the efficiency of healthcare services, and political will and community involvement. These real-world issues are beyond individual control (12). Therefore, although schistosomiasis awareness has been widely disseminated in many areas, the actual problem remains that people still cannot access safe water sources, adequate basic sanitation facilities, avoid exposure to contaminated water sources, or access sufficient medical resources (12, 53). Additionally, changing people’s perceptions of schistosomiasis is not a simple matter. For instance, in rural traditional communities, even long-term and repeated educational efforts on schistosomiasis may not significantly change the existing knowledge base in these communities (54).

In the long run, the impact of education is profound. Education can change human behavior. In a low-cost health education experiment for preventing schistosomiasis, elementary schools participating in the experiment actively encouraged students to value personal hygiene through teaching, such as using toilets, washing hands, and wearing shoes, and to be aware of the dangers of contaminated water. Following the education, their understanding of methods to prevent parasitic infections increased significantly, and they changed their behavioral habits (55). This experiment, being low-cost, sustainable, and replicable, was successfully implemented in other schools subsequently, demonstrating its feasibility (55).

Education methods need to be adjusted to accommodate different social backgrounds and age groups (56). For instance, healthcare workers and educators require more comprehensive and in-depth education, while ordinary community members can receive health education through community mobilization, and students can receive relevant education at school (53, 57). The forms of education are also diverse, and if schistosomiasis prevention education can be integrated into local culture and aligned with local social context, it can be better understood and accepted (12).

The benefits of education include improving individual awareness, helping people understand the causes, transmission routes, clinical symptoms, and preventive measures of schistosomiasis (58). Health education programs can enhance individual and community awareness, making it easier for people to perceive potential risk factors, take preventive measures, and actively seek early treatment (58). This can help prevent some patients from refusing diagnosis and treatment due to a lack of education and awareness, or fear, distrust, and other factors (59). Increasing patients’ understanding of the disease and medications through education may, to some extent, influence treatment adherence among the infected population (60). A deeper understanding of the disease can enhance patients’ willingness to seek treatment (61). Additionally, studies have found that among groups that have received health education, most women have stopped using water infected with schistosomes for laundry, and children actively reduce contact frequency, significantly reducing the rate of reinfection (62).

**Figure 6:**
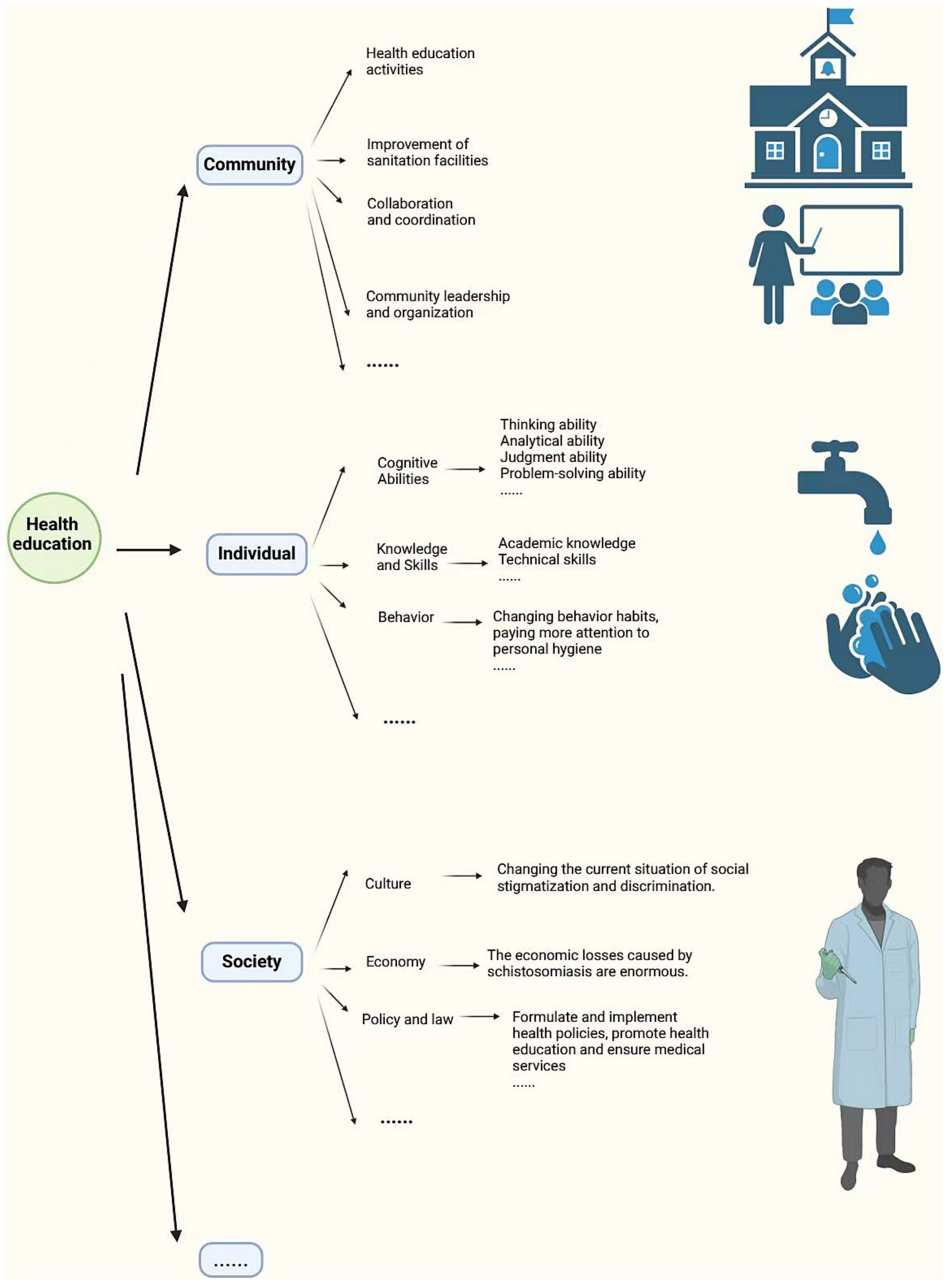
The role and impact of health education. Figure was created with BioRender.com.

In the past, traditional health education emphasized individual understanding of diseases, focusing on the simple transmission of knowledge and reading materials. Health literacy is a series of outcomes from health education and communication activities, but traditional health literacy no longer meets the demands of contemporary society (63). Modern health education models place greater emphasis on the ability to acquire knowledge, encourage community participation, and explore the political content of health education (63). Individual-focused health education has become a thing of the past, and behavioral research is increasingly shifting its focus to communities in an effort to address broader issues (12). Schools, as important communities, have a direct and effective impact on students. Therefore, schools implementing effective measures such as concentrated training for teachers, who can then teach knowledge one-to-many, allows students to gain more professional knowledge efficiently and ensures the accuracy and relevance of information (64) (Figure 6).

Patients with neglected tropical diseases, including schistosomiasis, are susceptible to social stigma and discrimination (65). These misconceptions often stem from a lack of understanding of schistosomiasis, low disease awareness, feelings of shame and embarrassment about being ill, avoidance and negative attitudes towards the disease, and even labeling and ridicule (66–68). Such stigma can affect employment, educational opportunities, interpersonal relationships, cause social isolation, and harm reputations (68, 69). Health education is a way to overcome stigma by spreading awareness of schistosomiasis, emphasizing the perspectives and attitudes of communities and patients, and enhancing understanding and recognition of schistosomiasis to reduce the number of patients, ultimately changing this stigmatized situation at its root (65).

Global health issues are interconnected, and to ensure the achievement of WHO goals, the Global Schistosomiasis Alliance (GSA) was established. The GSA calls for international cooperation to jointly address health inequities and rural poverty, striving to control schistosomiasis in countries and regions where the disease is endemic (70). As a platform, GSA can break down information barriers, coordinate cooperation between countries and regions, promote research and implementation, enhance efficiency and awareness, and facilitate the rational use of resources (70).

Comprehensive control requires long-term, continuous funding, but some international donors may lack a comprehensive understanding of the disease and programs, leading to funding being cut off before goals are achieved. Therefore, for policymakers and investors, health education on schistosomiasis may also be indispensable (61). Countries can also share knowledge and experience through education, working together to carry out health education activities and facilitate subsequent cooperation.

The strategies for the prevention and control of schistosomiasis have been refined through continuous practice. Currently, mass drug administration (MDA) with praziquantel has been proven to be an effective method (71). However, research into new drugs is necessary. If praziquantel loses its effectiveness due to resistance, and no alternative, more effective drugs are found, it will be challenging to sustain the current prevention and control outcomes (72). The reason for emphasizing these points is that education and technological progress are mutually reinforcing. We hope to plant a seed in people’s minds through education, accelerating scientific and technological development and promoting the emergence of innovative drugs and therapies. In summary, through health education, individuals can enhance their knowledge, develop good hygiene habits, and also increase social inclusivity and policy support, thereby effectively reducing the risk of infection. Education is not a quick fix; its impact requires long-term accumulation and the effort of several generations.

## 7 Impact, achievements, and challenges of health education

**Figure 7:**
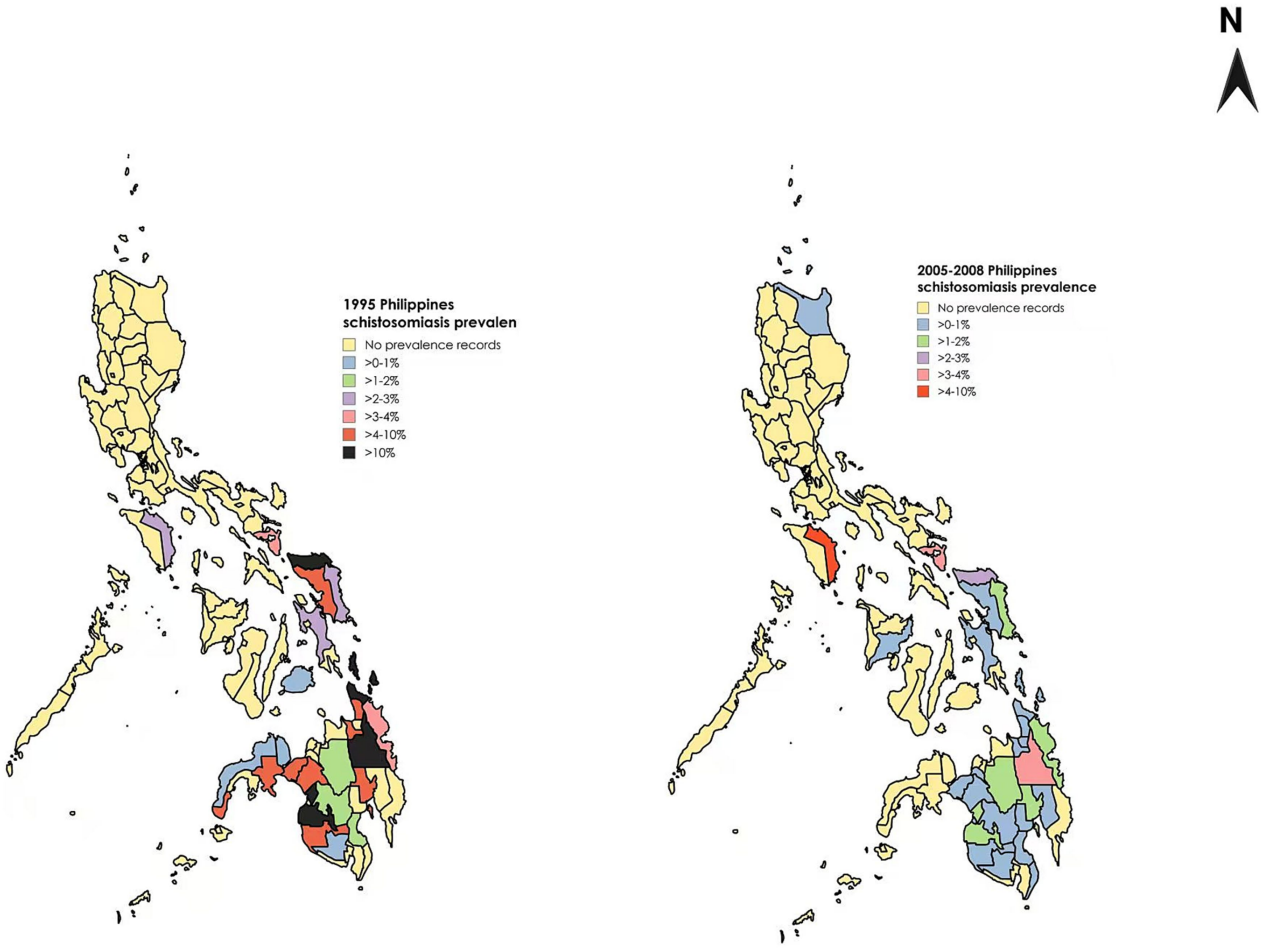
Comparison of schistosomiasis prevalence rates in different regions of the Philippines across various years. Figure was created with BioRender.com and MapChart.net.

In the past approximately 100 years, the Philippine government has implemented numerous measures to control and treat schistosomiasis (11). Particularly since the late 1970s, control efforts have been significantly strengthened with clear results (50). The application of praziquantel has led to a shift in the focus of control methods, the use of health education as an auxiliary measure, and a change in people’s mindset from treatment to prevention, all of which have greatly enhanced people’s awareness of and ability to prevent schistosomiasis (73). Data show that the prevalence rate of schistosomiasis in the Philippines was higher than 10% in 1990, around 5% in 1995, and approximately 1.3% in 2005–2008, and has further declined to about 1% in 2023 (74), demonstrating a continuous decline in prevalence and the effectiveness of a comprehensive approach (75) (Figure 7).

From a global perspective, there have been significant victories in the prevention and control of schistosomiasis, for instance, many countries and regions, such as Japan and China, have largely achieved control over schistosomiasis. The governance experiences of these regions are valuable and offer practical guidance for schistosomiasis prevention and control efforts (5, 76). However, the socioeconomic and ecological diversity in endemic regions necessitate the development of tailored, location-specific approaches. Countries with a high global burden of schistosomiasis, such as Nigeria and Tanzania, have also implemented comparable control strategies, including large-scale drug administration and health education campaigns. Nevertheless, the national average prevalence of schistosomiasis in these countries remains high. This is closely related to factors such as environmental policies, economy, and education, with various factors influencing each other and being closely interconnected. For example, improper management of water resources in environmental factors directly affects snail control; policy factors constrain the implementation of prevention and control efforts as well as scientific research; economic conditions lead to difficulties in the implementation of projects such as drug treatment and health education activities, or their inadequate actual implementation; and the insufficient coverage of health education results in many people lacking awareness of schistosomiasis and public health consciousness, making it even more challenging to implement prevention and control measures (77, 78). From the governance experiences of various countries, the overall governance approach exhibits a certain degree of similarity. However, its specific application has led to varying outcomes under different contexts. Therefore, when drawing lessons from successful experiences, greater attention should be paid to the differences arising from national conditions, with a focus on examining the practical applicability of these measures (79). Strengthening communication and cooperation among countries to promote resource sharing can significantly reduce duplication and waste. There have been many successful cases in recent years (80). In terms of education, countries and regions that have successfully managed schistosomiasis and have abundant experience can provide training to policymakers and professionals in schistosomiasis-endemic areas with less experience and offer them opportunities for visits (81).

Although significant progress has been made in the prevention and control of schistosomiasis in the Philippines, there are still many challenges (73, 82). Agriculture as a vital component of the Philippine economy, providing employment for a large portion of the population. As of 2020, approximately 47.4% of Filipinos still live in towns or rural areas, the large rural population is a notable group deserving of attention (83). In terms of geography, rural and coastal areas are schistosomiasis endemic areas, and these high-prevalence areas often share similar characteristics: lower economic and educational levels, poorer sanitary conditions, proximity to water sources, and residents who primarily rely on agriculture and fishing for their livelihoods (84). For those engaged in fishing, agriculture, and animal husbandry, occupational exposure makes them particularly susceptible to coming into contact with schistosomiasis, thereby increasing their likelihood of contracting the disease (85).

Climate change is increasingly posing a serious challenge to the sustainability of global schistosomiasis control efforts. This challenge is primarily manifested in rising global temperatures, shifting rainfall patterns, and the increased frequency of extreme weather events (86). In the Philippines, coastal and low-lying areas are particularly vulnerable to these changes, which may lead to alterations in the suitable habitats and population fluctuations of *Oncomelania hupensis*, the intermediate snail host, thereby impacting the dynamics of disease transmission (87). Similar trends have been observed in other endemic regions, such as East Africa, where climate change has been linked to the spread of *Schistosoma mansoni* (88). To address these challenges, it is essential to develop adaptive strategies and implement integrated control measures that take into account the transmission patterns of *Schistosoma japonicum*.

Due to the zoonotic nature of *Schistosoma japonicum*, with numerous intermediate and reservoir hosts, the implementation of prevention and control measures faces challenges. Additionally, the complex archipelagic terrain of the Philippines also increases the complexity of prevention and control efforts (3). At the same time, as a developing country, the relatively inadequate sanitation facilities and insufficient medical resources make it easier for people to come into contact with contaminated water sources (89). Another significant concern is the issue of resistance to the drug praziquantel. The search for alternative drugs and better medications is necessary, especially in the long run. Ignoring the importance of medication and failing to monitor the development of schistosomiasis could lead to serious consequences (90).

As a supplementary measure, although health education has been implemented for many years, it has not completely changed people’s perceptions. Strengthening the depth and breadth of health education remains a task for the future. At the same time, the effectiveness of education is largely contingent on people’ s access to basic WASH (Water, Sanitation, and Hygiene)services. In many developing countries, particularly in rural areas, the lack of access to clean water and sanitation facilities makes it difficult for people to take effective preventive measures, even if they possess the relevant knowledge (91). Studies from Ethiopia have illuminated that the dearth of basic WASH facilities in schools has compromised students’ health and their attendance rates, thereby curtailing the efficacy of education even further (92). Despite the implementation of educational programs and school-based deworming initiatives, the prevalence of schistosomiasis in Ethiopia remains high (93). In the survey conducted in Brazil, it was similarly found that improvements in water supply and sanitation facilities are crucial for controlling the transmission of schistosomiasis (94). With global climate change, access to water resources is being affected, and many neglected tropical diseases already impacted by the lack of WASH services will suffer even greater negative consequences as a result.

Therefore, to enhance the practical role of education in the control of schistosomiasis, it is essential to simultaneously improve access to WASH services. Only when people have access to clean drinking water and adequate sanitation facilities can education truly realize its potential in helping to reduce the transmission and infection of schistosomiasis (95, 96). This requires a concerted effort from both government and non-governmental organizations to ensure the widespread availability of WASH services and the effective implementation of educational programs.

## 8 Conclusion

This paper delves into the prevention and control measures for schistosomiasis and finds that within a comprehensive strategy for schistosomiasis control, education plays a critical role in the prevention and control of schistosomiasis in the Philippines. The paper extends the discussion on the significance of education. The implementation of systematic health education activities has played a complementary role in reducing schistosomiasis infection rates in the Philippines. These activities have not only enhanced public health awareness and promoted understanding of preventive measures and early treatment methods, but they have also increased people’s willingness and ability to take proactive preventive measures. Looking ahead, low-cost, high-return health education appears to be a promising measure. Further improving the content and diversifying the forms of education may provide added impetus to schistosomiasis prevention and control efforts in the Philippines.

## References

[1] WHO. (2025). Neglected tropical diseases. Available online at: https://www.who.int/news-room/questions-and-answers/item/neglected-tropical-diseases (Accessed January 8, 2025).

[2] Colley DG Secor WE. Immunology of human schistosomiasis. Parasite Immunol. (2014) 36:347–57. doi: 10.1111/pim.12087, PMID: 25142505 PMC4278558

[3] Díaz AV Walker M Webster JP. Reaching the World Health Organization elimination targets for schistosomiasis: the importance of a one health perspective. Philos Trans R Soc Lond Ser B Biol Sci. (2023) 378:20220274. doi: 10.1098/rstb.2022.0274, PMID: 37598697 PMC10440173

[4] Francisco I Jiz M Rosenbaum M Baltazar P Steele JA. Knowledge, attitudes and practices related to schistosomiasis transmission and control in Leyte, Philippines. PLoS Negl Trop Dis. (2019) 13:e0007358. doi: 10.1371/journal.pntd.0007358, PMID: 31048882 PMC6516667

[5] Rollinson D Knopp S Levitz S Stothard JR Tchuem Tchuenté L-A Garba A. Time to set the agenda for schistosomiasis elimination. Acta Trop. (2013) 128:423–40. doi: 10.1016/j.actatropica.2012.04.013, PMID: 22580511

[6] Delos Trinos J Wulandari LPL Clarke N Belizario V Jr Kaldor J Nery SV. Prevalence of soil-transmitted helminth infections, schistosomiasis, and lymphatic filariasis before and after preventive chemotherapy initiation in the Philippines: a systematic review and meta-analysis. PLoS Negl Trop Dis. (2021) 15:e0010026. doi: 10.1371/journal.pntd.0010026, PMID: 34928944 PMC8722724

[7] Leonardo L Rivera P Saniel O Villacorte E Lebanan MA Crisostomo B. A national baseline prevalence survey of schistosomiasis in the Philippines using stratified two-step systematic cluster sampling design. J Trop Med. (2012) 2012:936128. doi: 10.1155/2012/936128 22518170 PMC3306976

[8] Costain AH MacDonald AS Smits HH. Schistosome egg migration: mechanisms, pathogenesis and host immune responses. Front Immunol. (2018) 9:3042. doi: 10.3389/fimmu.2018.03042, PMID: 30619372 PMC6306409

[9] Costain AH MacDonald AS Smits HH. Corrigendum: Schistosome egg migration: mechanisms, pathogenesis and host immune responses. Front Immunol. (2019) 10:749. doi: 10.3389/fimmu.2019.00749, PMID: 31031753 PMC6470403

[10] Ross AGP Olveda RM Acosta L Harn DA Chy D Li Y. Road to the elimination of schistosomiasis from Asia: the journey is far from over. Microbes Infect. (2013) 15:858–65. doi: 10.1016/j.micinf.2013.07.010, PMID: 23973709 PMC4433715

[11] Blas BL Rosales MI Lipayon IL Yasuraoka K Matsuda H Hayashi M. The schistosomiasis problem in the Philippines: a review. Parasitol Int. (2004) 53:127–34. doi: 10.1016/j.parint.2004.01.003, PMID: 15081944

[12] Kloos H. Human behavior, health education and schistosomiasis control: a review. Soc Sci Med. (1995) 40:1497–511. PMID: 7667655 10.1016/0277-9536(94)00310-p

[13] Sokolow SH Huttinger E Jouanard N Hsieh MH Lafferty KD Kuris AM. Reduced transmission of human schistosomiasis after restoration of a native river prawn that preys on the snail intermediate host. Proc Natl Acad Sci USA. (2015) 112:9650–5. doi: 10.1073/pnas.1502651112, PMID: 26195752 PMC4534245

[14] Belizario VY de Cadiz AE Navarro RC Flores MJC Molina VB Dalisay SNM. The status of schistosomiasis japonica control in the Philippines: the need for an integrated approach to address a multidimensional problem. IJOH. (2022) 8:8–19. doi: 10.14202/IJOH.2022.8-19

[15] Concepcion M. B. (1970). Country profiles, the Philippines. The Population Council. Ford Foundation, New York, N.Y: The International Institute for the Study of Human Reproduction, Columbia University.

[16] Ministry of Foreign Affairs of the People's Republic of China. (2023). Philippines Country profile. Available online at: https://www.mfa.gov.cn/web/gjhdq_676201/gj_676203/yz_676205/1206_676452/1206x0_676454/ (Accessed January 8, 2025).

[17] Gordon CA Kurscheid J Williams GM Clements ACA Li Y Zhou X-N. Asian schistosomiasis: current status and prospects for control leading to elimination. Trop Med Infect Dis. (2019) 4:4. doi: 10.3390/tropicalmed4010040, PMID: 30813615 PMC6473711

[18] Zhu H-R Liu L Zhou X-N Yang G-J. Ecological model to predict potential habitats of Oncomelania hupensis, the intermediate host of Schistosoma japonicum in the mountainous regions, China. PLoS Negl Trop Dis. (2015) 9:e0004028. doi: 10.1371/journal.pntd.0004028, PMID: 26305881 PMC4549249

[19] Kuo Y-J Paras G Tagami T Yi C Aquino LJC Oh H. A compartmental model for Schistosoma japonicum transmission dynamics in the Philippines. Acta Trop. (2024) 249:107084. doi: 10.1016/j.actatropica.2023.107084, PMID: 38029954

[20] Olveda RM Gray DJ. Schistosomiasis in the Philippines: innovative control approach is needed if elimination is the goal. Trop Med Infect Dis. (2019) 4:66. doi: 10.3390/tropicalmed4020066, PMID: 31013917 PMC6631753

[21] Inobaya MT Olveda RM Tallo V McManus DP Williams GM Harn DA. Schistosomiasis mass drug administration in the Philippines: lessons learnt and the global implications. Microbes Infect. (2015) 17:6–15. doi: 10.1016/j.micinf.2014.10.006, PMID: 25448635

[22] Pinot de Moira A Fulford AJC Kabatereine NB Ouma JH Booth M Dunne DW. Analysis of complex patterns of human exposure and immunity to schistosomiasis mansoni: the influence of age, sex, ethnicity and IgE. PLoS Negl Trop Dis. (2010) 4:e820. doi: 10.1371/journal.pntd.0000820, PMID: 20856909 PMC2939029

[23] Carlton EJ Hubbard A Wang S Spear RC. Repeated Schistosoma japonicum infection following treatment in two cohorts: evidence for host susceptibility to helminthiasis? PLoS Negl Trop Dis. (2013) 7:e2098. doi: 10.1371/journal.pntd.0002098, PMID: 23505589 PMC3591324

[24] Dalton PR Pole D. Water-contact patterns in relation to Schistosoma haematobium infection. Bull World Health Organ. (1978) 56:417–26. PMID: 308406 PMC2395583

[25] Friedman JF Kanzaria HK Acosta LP Langdon GC Manalo DL Wu H. Relationship between Schistosoma japonicum and nutritional status among children and young adults in Leyte, the Philippines. Am J Trop Med Hyg. (2005) 72:527–33. doi: 10.4269/ajtmh.2005.72.527, PMID: 15891125

[26] Cabanacan-Salibay C Torres M Luyon-Tabo HA Salibay SP. Schistosomiasis in children in the Philippines: beyond health issues. J Pediatr Infect Dis Soc. (2017) 12:238–48. doi: 10.1055/s-0037-1603354, PMID: 40148104

[27] Ezeamama AE McGarvey ST Hogan J Lapane KL Bellinger DC Acosta LP. Treatment for Schistosoma japonicum, reduction of intestinal parasite load, and cognitive test score improvements in school-aged children. PLoS Negl Trop Dis. (2012) 6:e1634. doi: 10.1371/journal.pntd.0001634, PMID: 22563514 PMC3341324

[28] Gobbi F Tamarozzi F Buonfrate D van Lieshout L Bisoffi Z Bottieau E. New insights on acute and chronic schistosomiasis: do we need a redefinition? Trends Parasitol. (2020) 36:660–7. doi: 10.1016/j.pt.2020.05.009, PMID: 32505540

[29] Nour NM. Schistosomiasis: health effects on women. Rev Obstet Gynecol. (2010) 3:28. doi: 10.3909/riog0109 20508780 PMC2876318

[30] Han H Peng J Gobert GN Hong Y Zhang M Han Y. Apoptosis phenomenon in the schistosomulum and adult worm life cycle stages of Schistosoma japonicum. Parasitol Int. (2013) 62:100–8. doi: 10.1016/j.parint.2012.09.008, PMID: 23159324

[31] Faust EC Meleney HE. The life history of Schistosoma japonicum Katsurada. Chin Med J. (1923) 37:726–34.

[32] Department of Health Republic of the Philippines. (2015). Schistosomiasis control and elimination program. Available online at: https://ro9.doh.gov.ph/index.php/health-programs/infectious-disease-program/schistosomiasis-control-and-elimination-program (Accessed November 15, 2024).

[33] Department of Health Republic of the Philippines. (2023). Schistosomiasis control and elimination program. Available online at: https://doh.gov.ph/uhc/health-programs/schistosomiasis-control-and-elimination-program/ (Accessed November 15, 2024).

[34] Tenorio JCB Molina EC Development, Extension & Technology. Schistosoma japonicum in the Philippines: its epidemiology, diagnostics, control, and elimination. JARDET. (2021) 3:71–87. doi: 10.5281/zenodo.8296531

[35] Kumagai T Furushima-Shimogawara R Ohmae H Wang T-P Lu S Chen R. Detection of early and single infections of Schistosoma japonicum in the intermediate host snail, Oncomelania hupensis, by PCR and loop-mediated isothermal amplification (LAMP) assay. Am J Trop Med Hyg. (2010) 83:542–8. doi: 10.4269/ajtmh.2010.10-0016, PMID: 20810818 PMC2929049

[36] Notomi T Mori Y Tomita N Kanda H. Loop-mediated isothermal amplification (LAMP): principle, features, and future prospects. J Microbiol. (2015) 53:1–5. doi: 10.1007/s12275-015-4656-9, PMID: 25557475

[37] Ye YY Zhao S Liu YH Bi NN Dong X Xiong CR. Performance of a recombinase - aided amplification assay for detection of Schistosoma japonicum infections in Oncomelania hupensis. Zhongguo Xue Xi Chong Bing Fang Zhi Za Zhi. (2021) 33:185–8. doi: 10.16250/j.32.1374.2020281, PMID: 34008366

[38] Leonardo LR Rivera PT Crisostomo BA Sarol JN Bantayan NC Tiu WU. A study of the environmental determinants of malaria and schistosomiasis in the Philippines using remote sensing and geographic information systems. Parassitologia. (2005) 47:105–14. PMID: 16044679

[39] Olliaro PL Vaillant MT Belizario VJ Lwambo NJS Ouldabdallahi M Pieri OS. A multicentre randomized controlled trial of the efficacy and safety of single-dose praziquantel at 40 mg/kg vs. 60 mg/kg for treating intestinal schistosomiasis in the Philippines, Mauritania, Tanzania and Brazil. PLoS Negl Trop Dis. (2011) 5:e1165. doi: 10.1371/journal.pntd.0001165, PMID: 21695161 PMC3114749

[40] Cioli D Pica-Mattoccia L. Praziquantel. Parasitol Res. (2003) 90:S3–9. doi: 10.1007/s00436-002-0751-z, PMID: 12811543

[41] Molehin AJ. Schistosomiasis vaccine development: update on human clinical trials. J Biomed Sci. (2020) 27:28. doi: 10.1186/s12929-020-0621-y, PMID: 31969170 PMC6977295

[42] Merrifield M Hotez PJ Beaumier CM Gillespie P Strych U Hayward T. Advancing a vaccine to prevent human schistosomiasis. Vaccine. (2016) 34:2988–91. doi: 10.1016/j.vaccine.2016.03.079, PMID: 27036511

[43] You H Gobert GN Cai P Mou R Nawaratna S Fang G. Suppression of the insulin receptors in adult Schistosoma japonicum impacts on parasite growth and development: further evidence of vaccine potential. PLoS Negl Trop Dis. (2015) 9:e0003730. doi: 10.1371/journal.pntd.0003730, PMID: 25961574 PMC4427307

[44] Zumuk CP Jones MK Navarro S Gray DJ You H. Transmission-blocking vaccines against schistosomiasis japonica. Int J Mol Sci. (2024) 25:1707. doi: 10.3390/ijms25031707 38338980 PMC10855202

[45] McManus DP Bergquist R Cai P Ranasinghe S Tebeje BM You H. Schistosomiasis—from immunopathology to vaccines. Semin Immunopathol. (2020) 42:355–71. doi: 10.1007/s00281-020-00789-x, PMID: 32076812 PMC7223304

[46] Grimes JET Croll D Harrison WE Utzinger J Freeman MC Templeton MR. The roles of water, sanitation and hygiene in reducing schistosomiasis: a review. Parasit Vectors. (2015) 8:156. doi: 10.1186/s13071-015-0766-9 25884172 PMC4377019

[47] McCullough FS Gayral P Duncan J Christie JD. Molluscicides in schistosomiasis control. Bull World Health Organ. (1980) 58:681–9. PMID: 6975179 PMC2395986

[48] Wang W Qin Z Zhu D Wei Y Li S Duan L. Synthesis, bioactivity evaluation, and toxicity assessment of novel salicylanilide ester derivatives as cercaricides against Schistosoma japonicum and molluscicides against Oncomelania hupensis. Antimicrob Agents Chemother. (2016) 60:323–31. doi: 10.1128/AAC.01539-15, PMID: 26503661 PMC4704174

[49] Zhou L-Y Deng Y Steinmann P Yang K. The effects of health education on schistosomiasis japonica prevalence and relevant knowledge in the People's Republic of China: a systematic review and meta-analysis. Parasitol Int. (2013) 62:150–6. doi: 10.1016/j.parint.2012.11.006, PMID: 23201566

[50] Bizimana P Ortu G Van Geertruyden J-P Nsabiyumva F Nkeshimana A Muhimpundu E. Integration of schistosomiasis control activities within the primary health care system: a critical review. Parasit Vectors. (2019) 12:393. doi: 10.1186/s13071-019-3652-z, PMID: 31391100 PMC6686413

[51] Bishop HG. Menace of schistosomiasis: its true neglected nature in Nigeria. MOJPH. (2017) 6:186. doi: 10.15406/mojph.2017.06.00186

[52] Sacolo H Chimbari M Kalinda C. Knowledge, attitudes and practices on schistosomiasis in sub-Saharan Africa: a systematic review. BMC Infect Dis. (2018) 18:46. doi: 10.1186/s12879-017-2923-6, PMID: 29347919 PMC5773048

[53] Uchoa E Barreto SM Firmo JOA Guerra HL Pimenta FG Lima Costa MFF. The control of schistosomiasis in Brazil: an ethno–epidemiological study of the effectiveness of a community mobilization program for health education. Soc Sci Med. (2000) 51:1529–41. doi: 10.1016/S0277-9536(00)00052-6, PMID: 11077955

[54] Sow S de Vlas SJ Mbaye A Polman K Gryseels B. Low awareness of intestinal schistosomiasis in northern Senegal after 7 years of health education as part of intense control and research activities. Trop Med Int Health. (2003) 8:744–9. doi: 10.1046/j.1365-3156.2003.01080.x, PMID: 12869097

[55] Lansdown R Ledward A Hall A Issae W Yona E Matulu J. Schistosomiasis, helminth infection and health education in Tanzania: achieving behaviour change in primary schools. Health Educ Res. (2002) 17:425–33. doi: 10.1093/her/17.4.425, PMID: 12197588

[56] Gazzinelli MF Lobato L Andrade G Matoso LF Diemert DJ Gazzinelli A. Improving the understanding of schistosomiasis among adolescents in endemic areas in Brazil: a comparison of educational methods. Patient Educ Couns. (2016) 99:1657–62. doi: 10.1016/j.pec.2016.04.010, PMID: 27180618 PMC5028251

[57] WHO. (1990). Health education in the control of schistosomiasis. Available online at: https://iris.who.int/handle/10665/39567 (Accessed November 15, 2024).

[58] Anumudu CI Onile OS Awobode H Gboyega-Tokunbo A Oladele V Adebayo A. Socio-cultural and environmental determinants of a proposed schistosomiasis health education intervention in Eggua, Nigeria. JBH. (2019) 8:92–100. doi: 10.5455/jbh.20190521094753

[59] Ahlberg BM Mwangi R Poggensee G Feldermeier H Krantz I. Better infection than hunger'. A study of illness perceptions with special focus on urinary schistosomiasis in northern Tanzania. Afr Sociol Rev. (2003) 7:18–34. Available at: https://www.jstor.org/stable/24487375

[60] Jin J Sklar GE Min Sen Oh V Chuen Li S. Factors affecting therapeutic compliance: a review from the patient’s perspective. Ther Clin Risk Manag. (2008) 4:269–86. doi: 10.2147/tcrm.s1458, PMID: 18728716 PMC2503662

[61] Ross AGP Chau TN Inobaya MT Olveda RM Li Y Harn DA. A new global strategy for the elimination of schistosomiasis. Int J Infect Dis. (2017a) 54:130–7. doi: 10.1016/j.ijid.2016.09.023, PMID: 27939558

[62] Hu G-H Jia H Song K-Y Lin D-D Zhang J Cao C-L. The role of health education and health promotion in the control of schistosomiasis: experiences from a 12-year intervention study in the Poyang Lake area. Acta Trop. (2005) 96:232–41. doi: 10.1016/j.actatropica.2005.07.016, PMID: 16154103

[63] Nutbeam D. Health literacy as a public health goal: a challenge for contemporary health education and communication strategies into the 21st century. Health Promot Int. (2000) 15:259–67. doi: 10.1093/heapro/15.3.259

[64] Murta FLG Massara CL Rodrigues MG Beck LCNH Favre TC. Teachers as multipliers of knowledge about schistosomiasis: a possible approach for health education programmes. BMC Infect Dis. (2022) 22:853. doi: 10.1186/s12879-022-07829-x, PMID: 36376818 PMC9664691

[65] Hofstraat K van Brakel WH. Social stigma towards neglected tropical diseases: a systematic review. Int Health. (2016) 8:i53–70. doi: 10.1093/inthealth/ihv071, PMID: 26940310

[66] Musuva RM Awiti A Omedo M Ogutu M Secor WE Montgomery SP. Community knowledge, attitudes and practices on schistosomiasis in western Kenya-the SCORE project. Am J Trop Med Hyg. (2014) 90:646–52. doi: 10.4269/ajtmh.13-0488 24534810 PMC3973508

[67] Mwanga JR Magnussen P Mugashe TLCL Gabone TLRM Aagaard-Hansen J. Schistosomiasis-related perceptions, attitudes and treatment-seeking practices in Magu district, Tanzania: public health implications. J Biosoc Sci. (2004) 36:63–81. doi: 10.1017/S0021932003006114, PMID: 14989532

[68] Odhiambo GO Musuva RM Atuncha VO Mutete ET Odiere MR Onyango RO. Low levels of awareness despite high prevalence of schistosomiasis among communities in Nyalenda informal settlement, Kisumu City. Western Kenya *PLoS Negl Trop Dis*. (2014) 8:e2784. doi: 10.1371/journal.pntd.0002784, PMID: 24699502 PMC3974654

[69] Takougang I Meli J Fotso S Angwafo F Kamajeu R Ndumbe PM. Some social determinants of urinary schistosomiasis in northern Cameroon: implications for schistosomiasis control. Afr J Health Sci. (2004) 11:111–20. doi: 10.4314/ajhs.v11i3.30788, PMID: 17298128

[70] Savioli L Albonico M Colley DG Correa-Oliveira R Fenwick A Green W. Building a global schistosomiasis alliance: an opportunity to join forces to fight inequality and rural poverty. Infect Dis Poverty. (2017) 6:79–84. doi: 10.1186/s40249-017-0280-8 28330495 PMC5363045

[71] Wang W Liang Y. Mass drug administration (MDA) for schistosomiasis. J Infect Dis. (2015) 211:848–9. doi: 10.1093/infdis/jiu506, PMID: 25205633

[72] Bergquist R Utzinger J Keiser J. Controlling schistosomiasis with praziquantel: how much longer without a viable alternative? Infect Dis Poverty. (2017) 6:74. doi: 10.1186/s40249-017-0286-2, PMID: 28351414 PMC5371198

[73] Leonardo L Chigusa Y Kikuchi M Kato-Hayashi N Kawazu S Angeles JM. Schistosomiasis in the Philippines: challenges and some successes in control. Southeast Asian J Trop Med Public Health. (2016) 47:651–66.

[74] WHO. (2024). Control of neglected tropical diseases. Available online at: https://www.who.int/teams/control-of-neglected-tropical-diseases/data-platforms/pct-databank/schistosomiasis (Accessed November 15, 2024).

[75] Inobaya MT Chau TN Ng SK MacDougall C Olveda RM Tallo VL. Mass drug administration and the sustainable control of schistosomiasis: an evaluation of treatment compliance in the rural Philippines. Parasit Vectors. (2018) 11:441. doi: 10.1186/s13071-018-3022-2, PMID: 30064469 PMC6069569

[76] Zhang L He J Yang F Dang H Li Y Guo S. Progress of schistosomiasis control in People's Republic of China in 2022. Zhongguo Xue Xi Chong Bing Fang Zhi Za Zhi. (2023) 35:217–24. doi: 10.16250/j.32.1374.2023073, PMID: 37455091

[77] Oyeyemi OT de Jesus Jeremias W Grenfell RFQ. Schistosomiasis in Nigeria: gleaning from the past to improve current efforts towards control. One Health. (2020) 11:100183. doi: 10.1016/j.onehlt.2020.100183, PMID: 33072838 PMC7553878

[78] Stothard JR Chitsulo L Kristensen TK Utzinger J. Control of schistosomiasis in sub-Saharan Africa: progress made, new opportunities and remaining challenges. Parasitology. (2009) 136:1665–75. doi: 10.1017/S0031182009991272, PMID: 19814845

[79] Liang S Abe EM Zhou X-N. Integrating ecological approaches to interrupt schistosomiasis transmission: opportunities and challenges. Infect Dis Poverty. (2018) 7:124. doi: 10.1186/s40249-018-0506-4 30541611 PMC6291957

[80] Song L-G Wu X-Y Sacko M Wu Z-D. History of schistosomiasis epidemiology, current status, and challenges in China: on the road to schistosomiasis elimination. Parasitol Res. (2016) 115:4071–81. doi: 10.1007/s00436-016-5253-5, PMID: 27679451

[81] Xu J Yu Q Tchuenté L-AT Bergquist R Sacko M Utzinger J. Enhancing collaboration between China and African countries for schistosomiasis control. Lancet Infect Dis. (2016) 16:376–83. doi: 10.1016/S1473-3099(15)00360-6, PMID: 26851829

[82] Fenwick A Rollinson D Southgate V. Implementation of human schistosomiasis control: challenges and prospects. Adv Parasitol. (2006) 61:567–622. doi: 10.1016/S0065-308X(05)61013-5, PMID: 16735173

[83] Raihan A. The dynamic nexus between economic growth, renewable energy use, urbanization, industrialization, tourism, agricultural productivity, forest area, and carbon dioxide emissions in the Philippines. Energy Nexus. (2023) 9:1001. doi: 10.1016/j.nexus.2023.100180

[84] Ross AGP Olveda RM McManus DP Harn DA Chy D Li Y. Risk factors for human helminthiases in rural Philippines. Int J Infect Dis. (2017b) 54:150–5. doi: 10.1016/j.ijid.2016.09.025, PMID: 27717859

[85] Raso G Li Y Zhao Z Balen J Williams GM McManus DP. Spatial distribution of human Schistosoma japonicum infections in the Dongting Lake region, China. PLoS One. (2009) 4:e6947. doi: 10.1371/journal.pone.0006947, PMID: 19759819 PMC2736371

[86] Yang G-J Utzinger J Zhou X-N. Interplay between environment, agriculture and infectious diseases of poverty: case studies in China. Acta Trop. (2015) 141:399–406. doi: 10.1016/j.actatropica.2013.07.009, PMID: 23906612 PMC7117482

[87] Recopuerto-Medina LM Gutierrez FCU San Diego JAS Alviar NAE Santos JRM Dagamac NHA. MaxEnt modeling of the potential risk of schistosomiasis in the Philippines using bioclimatic factors. Parasitol Int. (2024) 98:102827. doi: 10.1016/j.parint.2023.102827, PMID: 38030120

[88] McCreesh N Nikulin G Booth M. Predicting the effects of climate change on *Schistosoma mansoni* transmission in eastern Africa. Parasit Vectors. (2015) 8:4–9. doi: 10.1186/s13071-014-0617-0, PMID: 25558917 PMC4297451

[89] Miranda GS Rodrigues JGM Silva JKADO Camelo GMA Silva-Souza N Neves RH. New challenges for the control of human schistosomiasis: the possible impact of wild rodents in *Schistosoma mansoni* transmission. Acta Trop. (2022) 236:106677. doi: 10.1016/j.actatropica.2022.106677, PMID: 36063905

[90] Doenhoff MJ Pica-Mattoccia L. Praziquantel for the treatment of schistosomiasis: its use for control in areas with endemic disease and prospects for drug resistance. Expert Rev Anti-Infect Ther. (2006) 4:199–210. doi: 10.1586/14787210.4.2.199, PMID: 16597202

[91] Dhital SR Chojenta C Loxton D. Multi-level factors associated with utilization of water, sanitation and hygiene services by mothers in Nepal. PLoS One. (2024) 19:e0283379. doi: 10.1371/journal.pone.0283379 38507421 PMC10954160

[92] Sharma MK Adhikari R Khanal SP Acharya D van Teijlingen E. Do school water, sanitation, and hygiene facilities affect students' health status, attendance, and educational achievements? A qualitative study in Nepal. Health Sci Rep. (2024) 7:e2293. doi: 10.1002/hsr2.2293 39131595 PMC11310280

[93] Deribew K Yewhalaw D Erko B Mekonnen Z. Urogenital schistosomiasis prevalence and diagnostic performance of urine filtration and urinalysis reagent strip in schoolchildren, Ethiopia. PLoS One. (2022) 17:e0271569. doi: 10.1371/journal.pone.0271569, PMID: 35877771 PMC9312429

[94] Santos MCS Oliveira GL Mingoti SA Heller L. Effect of environmental factors in reducing the prevalence of schistosomiasis in schoolchildren: an analysis of three extensive national prevalence surveys in Brazil (1950-2018). PLoS Negl Trop Dis. (2023) 17:e0010804. doi: 10.1371/journal.pntd.0010804, PMID: 37459358 PMC10374055

[95] Girmay AM Mengesha SD Dinssa DA Alemu ZA Wagari B Weldegebriel MG. Access to water, sanitation and hygiene (WASH) services and drinking water contamination risk levels in households of Bishoftu town, Ethiopia: a cross-sectional study. Health Sci Rep. (2023) 6:e1662. doi: 10.1002/hsr2.1662, PMID: 37920657 PMC10618431

[96] Li S Gong A Yin Y Su Q. Spatiotemporal characteristics and socioeconomic inequalities in water, sanitation, and hygiene access in China from 2000 to 2020: analysis of data from three national censuses. BMC Public Health. (2024) 24:3250. doi: 10.1186/s12889-024-20739-8, PMID: 39578807 PMC11583406

